# A Study on the Low-Intensity Cracking Resistance of Drainage Asphalt Mixtures by Graphene/Rubber Powder Compound Modified Asphalt

**DOI:** 10.3390/ma18153451

**Published:** 2025-07-23

**Authors:** Jingcheng Chen, Yongqiang Cheng, Ke Liang, Xiaojian Cao, Yanchao Wang, Qiangru Shen

**Affiliations:** 1 School of Transportation and Civil Engineering, Nantong University, Nantong 226019, China; jcchen2000@163.com (J.C.); yanchaowang1989@ntu.edu.cn (Y.W.); 2 Shanghai Zhenhua Heavy Industries Company Limited, Shanghai 224005, China; 3 Nantong Construction Engineering Quality Testing Center, Nantong 226000, China; 4 Department of BIM Reserach, Nantong Institute of Technology, Nantong 226002, China

**Keywords:** graphene/rubber powder composite modified asphalt, semicircular bending test, low-temperature crack resistance, microstructure, drained asphalt mixtures

## Abstract

In order to investigate the influence of graphene/rubber powder compound modified asphalt on the low-temperature cracking resistance of drainage asphalt mixtures, graphene/rubber powder compound modified asphalt mixtures were prepared using graphene/rubber powder compound modified asphalt for drainage asphalt mixtures, and compared with SBS-modified asphalt and rubber powder-modified asphalt, and the low-temperature cracking resistance of graphene/rubber powder compound modification asphalt mixtures was investigated through the Marshall Stability Test, Semi-circular Bending Test (SCB), and Freeze–Thaw Split Test. Research was carried out. At the same time, a scanning electric microscope (SEM) was adopted to analyze the micro-mechanism of the graphene/rubber powder compound modified asphalt mixtures under the microscopic condition. The findings showed that graphene dispersed the aggregation of rubber powder effectively in the microscopic state and improved the stability of the composite modified asphalt. The addition of graphene improved the fracture energy of rubber powder composite modified asphalt by 15.68% under the condition of −15 °C to 0 °C, which effectively slowed down the decrease of fracture energy; at −15 °C and −10 °C, the largest stresses were improved by 7.50% and 26.71%, respectively, compared to the drainage asphalt mixtures prepared as rubber powder-modified asphalt and SBS-modified asphalt. After a freeze–thaw cycle, the maximum stress decrease of graphene/rubber powder compound modified asphalt was 21.51% and 10.37% at −15 °C and 0 °C, respectively. When compared to rubber powder-modified asphalt, graphene/rubber powder compound modified asphalt significantly improved the low-intensity cracking resistance of drainage asphalt mixtures at low temperatures, slowed down the decrease of the maximum stress, and its low-temperature cracking resistance was more stable.

## 1. Introduction

Drainage asphalt mixtures are characterized by large voids and a high water permeability, but the viscosity of the asphalt is required to be higher because its mineral gradation mainly consists of coarse aggregates with a small proportion of fine aggregates and fillers. Graphene has become a global research hotspot in recent years because of its ultra-high strength, excellent conductivity, thermal conductivity, and chemical stability. Graphene is compounded with rubber powder as a modifier. Rubber powder improves the low-temperature performance of asphalt through elastic toughening, while graphene improves the high-temperature stability through the lamellar barrier effect, and the synergistic effect is significant. The road properties of asphalt are enhanced to some degree by the use of rubber powder prepared with discarded tires and incorporated into the matrix asphalt as a modifier [1,2,3]. Wang, XiaoPing et al. [4] and Ma, Qingwei et al. [5] tested and characterized the modification mechanism of rubber powder and its influence on the structure and performance of rubber powder/asphalt, and discovered the asphalt inclusion of rubber powder significantly increased the low-temperature cracking resistance and stability of mixtures, but the stability was not good, and segregation easily occurred in the preparation of modified asphalt, which restricted the large-scale application of rubber powder-modified asphalt in the rubber-modified asphalt in the large-scale application of roadbed pavement engineering.

Graphene, as a new type of nanomaterials, is characterized by high performance, a high specific surface area, and possesses excellent interfacial effects [6,7,8], and the application of graphene in rubber powder-modified asphalt can significantly enhance the comprehensive properties of modified asphalt. Xie, Yutong et al. [9] and Liao, Meijie et al. [10] found that graphene significantly improved the storage stability of rubber powder by means of a storage stabilization test, the testing of a dynamic shear rheometer (DSR) and a fluid microscopy (FM) test, in which fewer layers of graphene were more effective in improving the storage performance of rubber powder. Wu Zhiheng et al. [11] found that porous graphene was used as an additive in SBS/CR blend-modified asphalt, which improved the compatibility of SBS and asphalt through its nano pore structure, and significantly improved the high-temperature rutting resistance, low-temperature crack resistance, and fatigue resistance. It destroyed the original colloidal structure through the cooperation of electrophilic adsorption and non-porous chemical bonds, and promoted the uniform formation of the SBS network and CR swelling. Abbas Mukhtar Adnan and others [12] found that the addition of graphene can moderate the viscosity of rubber powder-modified asphalt and has a good compatibility with asphalt, which solves the problems of poor compatibility and poor construction performance of TRP-modified asphalt caused by rubber segregation, and improves the low-temperature crack resistance and reduces the degree of phase separation. Researchers have investigated the various performances of graphene/rubber powder compound modified asphalt, laying the foundation for the use of graphene/rubber powder compound modification in asphalt mixtures.

Yong, P. et al. [13] and Guo, Rui et al. [14] investigated the effect of graphene/rubber powder compound modified asphalt on the performance in SMA-13 mixtures, and found that graphene/rubber powder compound modified asphalt SMA-13 mixtures had the best heat and water stability, followed by SBS-modified asphalt mixtures and matrix asphalt mixtures, and the use of graphene modifiers enhanced the asphalt–aggregate adhesion, whose asphalt possesses a favorable thickening and viscosity. Liu, Yunfang et al. [15] explored the enhancement of graphene on asphalt road performance and its mechanism, the use of DMSO pretreated graphene to add to the 70# road petroleum asphalt, and found that the inclusion of graphene makes asphalt hardened, reduces the roughness of the surface of graphene-modified asphalt, and that it is prone to stress concentration, which reduces the mechanical properties and the resistance to deformation. Wu, Jin-rong et al. [16] found that voids and connected voids are the primary factors influencing the drainage and cryogenic properties of asphalt pavements. Li, Jing et al. [17] and Farouk et al. [18] used GO and SBS particles as compound modifiers for the compound modification of 70~# matrix asphalt, and found that the modification effect of GO on matrix asphalt was superior to that of modified asphalt. Liu, Binqing et al. [19] and Kong et al. [20] investigated the influence of graphene on the road property of rubber powder-modified asphalt mixtures, and found that rubber-modified asphalt was more stable after the inclusion of graphene. Related research has been carried out to test the individual performance of asphalt mixtures [21,22], but the exploration of graphene/rubber powder compound modified asphalt on the cryogenic cracking resistance of drainage asphalt mixtures is still insufficient.

In order to further study the low-temperature crack resistance of graphene/rubber powder compound modified asphalt on drainage asphalt mixtures [11], this paper prepares drainage asphalt mixtures of graphene/rubber powder compound modified asphalt, rubber powder-modified asphalt, and SBS-modified asphalt, and adopts the Marshall stability test, semicircular bending test (SCB), and freezing and thawing cleavage test to study the low-temperature crack resistance of graphene/rubber powder compound modified asphalt mixtures. The low-temperature cracking resistance of graphene/rubber powder compound modified asphalt was investigated by using the Marshall stability test, semicircular bending test (SCB), freeze–thaw splitting test [23], and at the same time, in order to study the fly-away of graphene in rubber powder-modified asphalt, the micro-morphology of graphene/rubber powder compound modified asphalt was observed by the SEM electron microscope scanning test, and the low-temperature cracking resistance of graphene/rubber powder compound modified asphalt was investigated for drainage asphalt mixes.

## 2. Test Materials and Methods

### 2.1. Test Materials

In this article, 70# A-rated road matrix asphalt was selected as the matrix asphalt for the production of graphene/rubber powder compound modified asphalt, rubber powder-modified asphalt and SBS-modified asphalt, and technical requirements refer to the code for the design of highway asphalt pavement (JTG d50-2017), as shown in Table 1.

**Figure 1:**
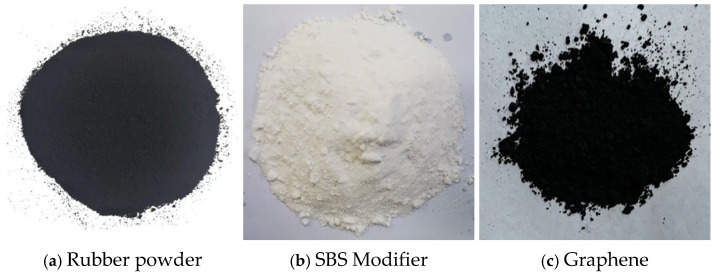
Modified materials.

Rubber powder from Hebei Hongyun Building Materials Co., Ltd. (Handan, China) was used to provide a particle size of 40 mesh, the appearance of no impurities, and graphene for the production of carbon abundance with the relevant performance and technology indexes as indicated in Table 2. For the SBS modifier by the Dongguan City, Zhenhua Engineering Plasticization Co., Ltd. production, three modified materials’ morphology are shown in Figure 1.

Basalt was used as the mineral aggregate for coarse aggregate. Refer to technical specifications for the construction of highway asphalt pavement (JTG F40-2017) for technical indicators, as shown in Table 3. Limestone was used for the fine concentrate, with a relative surface diameter of 2.77 g/m^3^, and limestone powder was used for the mineral powder, with a specific surface area of 482 m^3^/kg and a relative surface density of 2.69 g/cm^3^.

### 2.2. The Preparation of Modified Bitumen

**Figure 2:**
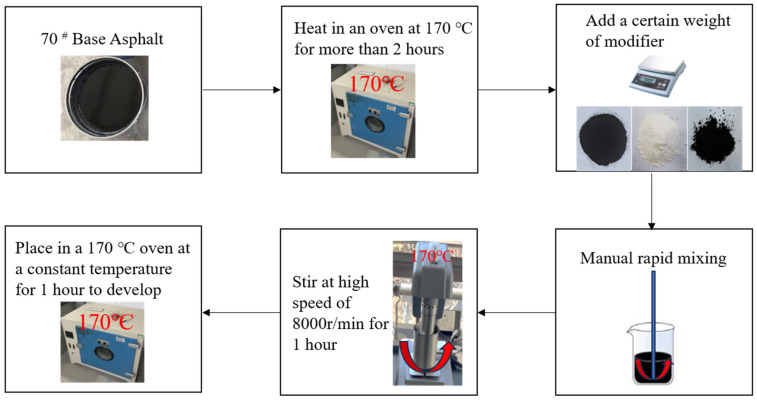
The preparation process of modified asphalt.

As illustrated in Figure 2 for the preparation process of modified asphalt, 70 base asphalt must be heated at 170 °C in the oven for more than 2 h to the flow state, according to the 10% of the external mixing amount of rubber powder into the asphalt. It then needs mixing, and then at a height of 170 °C, it undergoes a high-speed shear for more than 1 h, after which it must be placed in the oven at a constant temperature of 170 °C to develop for 1 h, for the preparation of rubber-modified asphalt (AR). Using the same method, 4% of the SBS modifier was included into the matrix asphalt, and SBS-modified asphalt was prepared according to the same method. It has been shown that the dosage of graphene is generally not more than 0.5% [6,7], and the main index that affects the bonding of large-void drainage asphalt mixtures between aggregates and asphalt is the rotational viscosity at 60 °C. Different contents of graphene are added to 10% rubber powder-modified asphalt, and when the dosage of graphene in 10% rubber powder-modified asphalt is 0.12%, the dynamic viscosity reaches a peak value at 60 °C, and the continued inclusion of graphene at this time will result in a decrease in the dynamic viscosity under 60 °C [24,25,26,27]. In the graphene composite modification stage, 0.12% graphene was pretreated with DMSO solvent in advance, and then mixed with rubber powder-modified asphalt. It was developed at 170 °C for 1 h. During this period, the dynamic viscosity at 60 °C was monitored with a rotary viscometer every 15 min until the viscosity reached the peak. In this paper, the dynamic viscosity at 60 °C is used as an index for making drainage asphalt mixtures, and a 0.12% dosing of graphene is used to create graphene/rubber powder compound modified asphalt, and at the same time, in order to improve the inclusion of graphene and asphalt compatibility, graphene is pretreated by dimethylsulfoxide (DMSO) solvent prior to entering into the asphalt [27,28,29], and 0.12% of graphene is added with 10% of the rubber powder into the substrate asphalt to prepare graphene/rubber powder compound modified asphalt (GO/AR) by the method shown in Figure 2.

### 2.3. Drainage Asphalt Mixture Grade Selection

**Figure 3:**
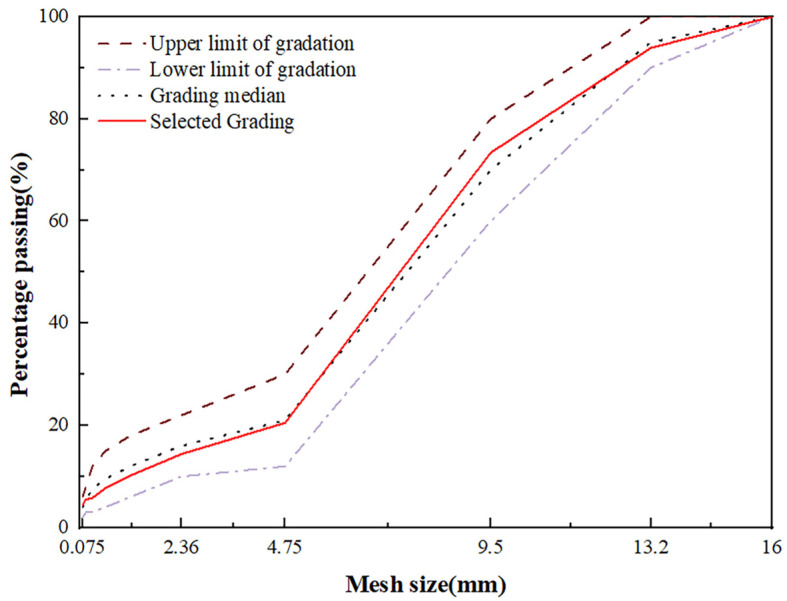
The grading selection of drainage asphalt mixtures.

Based on the “asphalt and asphalt mixture test specification for highway engineering” (JTG E20-2011), the drainage asphalt mixture gradation requirements and the mineral material gradation selection is shown in Figure 3. Through the mineral grade selection using Formula (1) for calculation, the oil–rock ratio is 4.87%.

(1) $$P_{b}=h\times A$$

(2) $$A=(2+0.02a+0.04b+0.08c+0.14d+0.3e+0.6f+1.6g)/48.74(m^{2}/\mathrm{kg})$$

Here, P_b_ is the oil–rock ratio; h is the thickness of the asphalt film, taking 14 μm; *A* is the total surface area of the aggregate; *a*, *b*, *c*, *d*, *e*, *f*, and *g* are the percentage of the passage of 4.75, 2.36, 1.18, 0.6, 0.3, 0.15, and 0.075 mm sieve holes, respectively.

The properties were tested for rubber powder-modified asphalt, SBS-modified asphalt, and graphene/rubber powder-modified asphalt, respectively, and the test methods are presented in Table 4.

### 2.4. The Fracture Energy Evaluation of Low-Temperature Crack Resistance

The cracking capacity of drainage asphalt mixtures at low operating temperatures is usually assessed on the basis of the fracture energy values when performing SCB tests [30,31,32]. The fracture energy is directly related to the test temperatures, and when the same type of drained asphalt mixes are subjected to SCB tests at different temperature conditions, the greater the test temperature, the increase in the fracture energy. When making calculations, a higher fracture energy means that the specimen has a good resistance to cracking at a given temperature. This fracture energy is determined by dividing the area enclosed by the loading–displacement profile and the *x*-axis during test loading by the area of the fracture surface of the specimen, as shown in Equations (3)–(5):

(3) $$G_{f}=\frac{W_{f}}{A_{realig}}$$

(4) $$W_{f}=\int Pdu$$

(5) $$A_{realig}=L\times t$$

where *G*_f_ is the rupture energy; *W_f_* is the load–displacement curve and the *X*-axis enclosed area, that is, the external force to do the work; *P* is the applied load; *A_realig_* is the area of the fracture surface of the specimen; *L* is the length of the toughness zone; and *t* is the specimen thickness.

**Figure 4:**
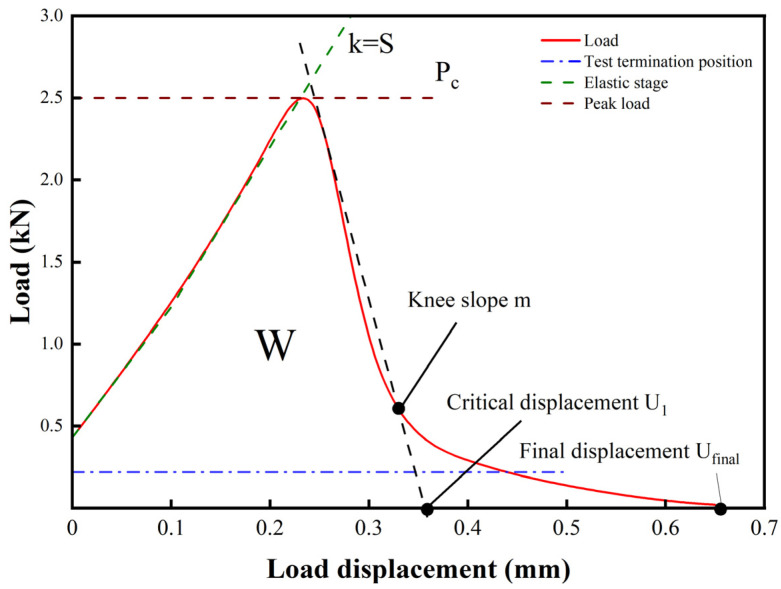
SCB loading curve.

As illustrated in Figure 4, the surface area under the experimental loading curve is the fracture work W, from which the fracture energy can be calculated, and the slope k of the elastic extremity on the curve is the rigidity S of the material.

### 2.5. Low-Temperature Cracking Resistance Test

For the low-thermal cracking performance testing for asphalt mixtures, the current commonly adopted testing methods are the trabecular bending test (3PBB), compact tensile test (DCT), individual tension test (IDT), and semicircular buckling test (SCB), etc. [33,34], and the commonly used evaluation indexes are the destructive strain, fracture energy, and freezing-section temperature [35]. In this paper, AASHTO TP105, a protocol developed by the American Highway Association for the SCB test, was adopted to test the low-intensity cracking properties of drainage asphalt mixtures.

**Figure 5:**
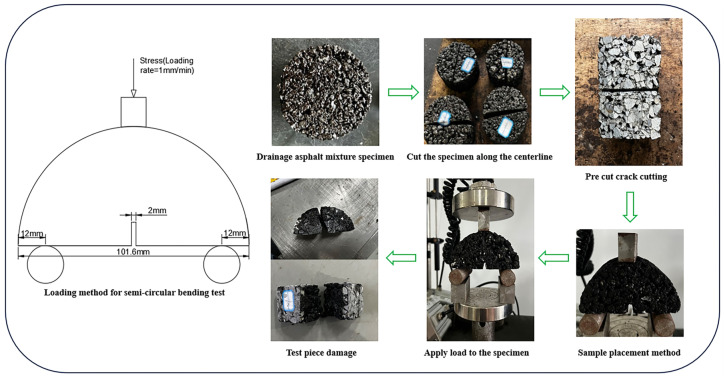
The SCB loading method and test process.

Specimens used in the semicircular bending test were standard hemicircular specimens of 101.6 mm in diameter and 63.5 mm in thickness, and a pre-sliced crack of 2 mm in width was cut in the middle of the specimen [36,37]. In view of reducing the error of the test to the final results, the loading temperature during the test should be as slow as possible. This test was performed using an electronic university testing machine, and the test loading rate was 1 mm/min. Taking into account the limiting minimum pavement temperature of the asphalt mixture [33], as well as with reference to the test temperature during the test of the low-temperature performance test of asphalt in the protocol “AASHTO TP105”, at the same time, based on the typical climate characteristics in China’s alpine regions and the actual needs of road engineering, the temperature range from −15 °C to 0 °C is focused on, which covers the transition threshold range with the highest risk of the low-temperature cracking of asphalt pavement. The final test temperatures are 0 °C, 5 °C, 10 °C, and 15 °C, respectively. Four specimens are taken for a parallel test at each temperature, and the test results are taken as the average value. The SCB loading mode and test process are shown in Figure 5.

### 2.6. The Low-Temperature Crack Resistance Test After Freeze–Thaw Cycles

In order to better analyze the cold-temperature crack resilience of drainage asphalt mixtures, combined with actual engineering asphalt mixture pavements in low-temperature environments [38,39], this paper tests the stress changes of standard semicircular specimens after one freeze–thaw cycle [40] to synthesize the evaluation of the cold-temperature anti-cracking performance of drainage asphalt mixtures prepared from graphene/rubber powder compound modified asphalt [41].

Keep the standard semicircular specimen with pre-cut cracks at 97.3–98.7 kPa for 15 min, then return to normal pressure and place it in water at 25 °C for 0.5 h. Then, take out the specimen and put it into a plastic bag, add 10 mL of water, and then put the specimen into the refrigerator at 0 °C, −5 °C, −10 °C, and −15 °C for 16 h, and then immediately put it into warm water at 60 °C and keep it warm for 24 h. After taking out the test, put it at room temperature to dry naturally, and then put it into 0 °C, −5 °C, −10 °C, and −15 °C to freeze for 16 h, and then pressurize the semicircular specimen with an electronic omnipotent testing apparatus at a rate of 1 mm/min to get the maximum stress that the specimen can withstand.

## 3. Experimental Results and Data Analysis

### 3.1. Marshall Stability

**Figure 6:**
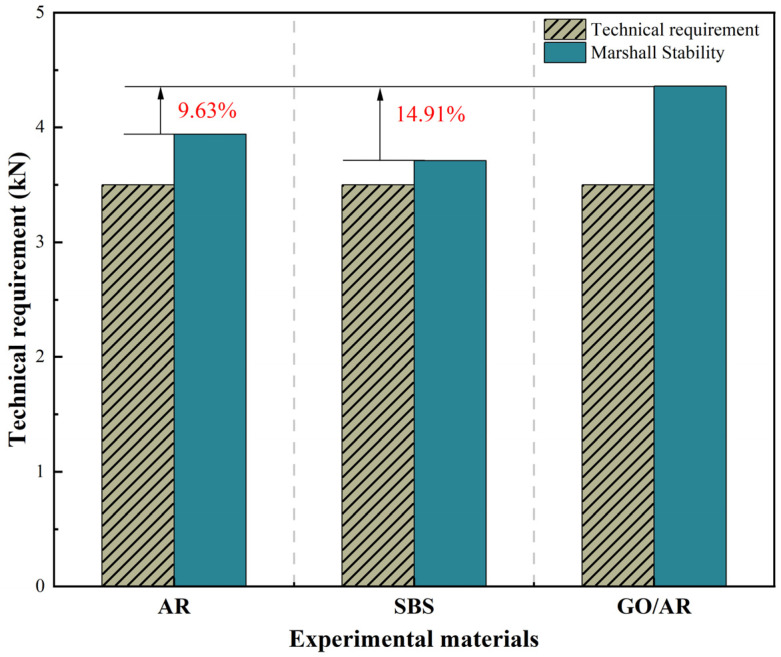
The Marshall stability of drainage asphalt mixtures.

The preparation of Marshall samples involved a series of chosen drainage asphalt mixtures, with the modified asphalt mixtures having an oil/stone ratio of 4.87%. The standard Marshall samples were immersed in water at 60 °C for testing in a thermostatic water bath for half an hour, with the outcomes displayed in Figure 6.

As shown in Figure 6, the Marshall stability of the three kinds of modified asphalt are above the technical standard of drainage asphalt mixtures. The average Marshall stability of graphene/rubber powder-modified asphalt is 4.36 kN, compared with the Marshall stability of the rubber powder-modified asphalt of 3.94 kN. The stability is improved by 9.63%, and the inclusion of graphene has changed the mechanism of the rubber powder-modified asphalt, which makes the modified asphalt with the degree of adhesion in the mixture better. In comparison with the SBS-modified asphalt of 3.71 kN, the graphene/rubber powder compound modified asphalt stability increased by 14.91%, which is more suitable to be utilized in drainage asphalt mixtures.

### 3.2. Fracture Energy at Different Temperatures

**Figure 7:**
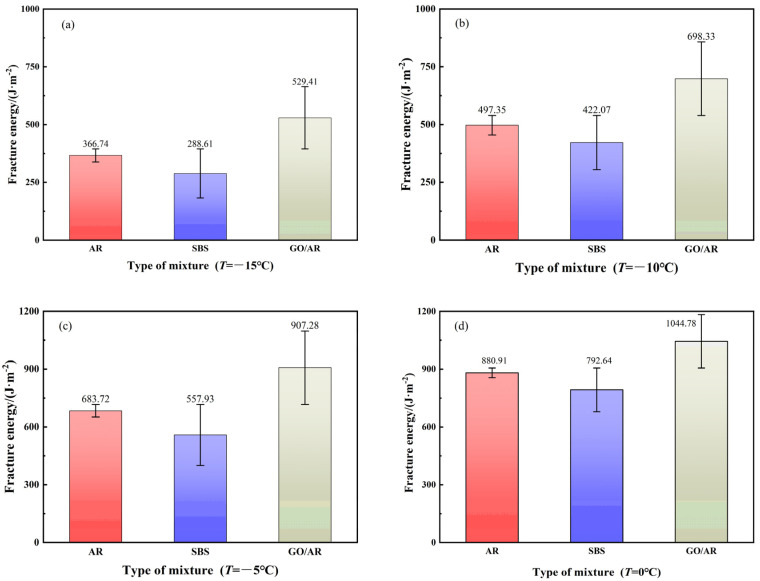
The fracture energy of drainage asphalt mixtures at different temperatures: (**a**) The experimental temperature is −15 °C. (**b**) The experimental temperature is −10 °C. (**c**) The experimental temperature is −5 °C. (**d**) The experimental temperature is 0 °C.

The energy required to break rubber powder-modified asphalt, SBS-modified asphalt, and graphene/rubber powder composite modified asphalt at varying low temperatures was determined using Equations (3) and (4), in that order, at temperatures of 0 °C, −5 °C, −10 °C, and −15 °C, with the outcomes depicted in Figure 7.

It can be seen from Figure 7 that with the increase of experimental temperatures, the fracture energy also increases, and the ductility of the permeable asphalt mixture gradually increases [39,41]. All data are calculated based on three groups of parallel tests, and the error bar represents the standard deviation. At T = 0 °C, the fracture energy of rubber powder-modification asphalt was 10.02% more than that of SBS-modification asphalt, while the inclusion of graphene enhanced the fracture energy of rubber powder-modification asphalt by 15.68%. As the temperature decreases, the fracture energy from rubber powder-modification asphalt was 21.30% higher than that of SBS-modification asphalt at T = −15 °C, which indicates that the SBS-modified asphalt has a worse low-temperature cracking resistance at lower temperatures. For modified asphalt, the overall fracture energy of rubber powder-modified asphalt decreased by 58.37% from T = 0 °C to T = −15 °C, the fracture energy of SBS-modified asphalt decreased by 63.59%, and graphene/rubber powder-modified asphalt fracture energy decreased by 49.33%. The inclusion of graphene decreased the degree of fracture energy decrease of rubber powder-modified asphalt, changed its physicochemical properties, and resulted in a better low-temperature crack resistance of modified asphalt.

### 3.3. The Low-Temperature Cracking Resistance of SCB Tests

**Figure 8:**
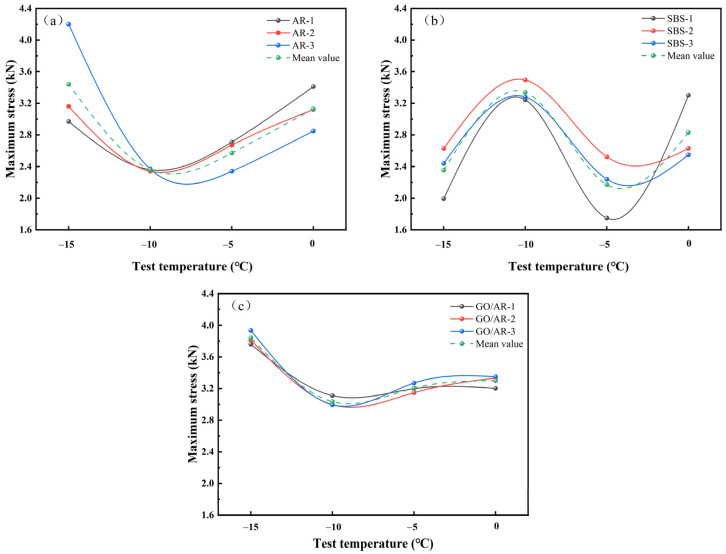
The maximum stress of SCB specimens at different temperatures: (**a**) Variation of AR maximum stress with temperature. (**b**) Variation of SBS maximum stress with temperature. (**c**) Variation of GO/AR maximum stress with temperature.

Standard semicircular specimens at different low temperatures were pressure tested and analyzed for maximum stress, and the stress testing results are illustrated in Figure 8.

Here, (a), (b), and (c) in Figure 8 show the compression of three groups of standard semicircular specimens prepared by three different modified asphalts at various temperatures, respectively. It can be observed through Figure 8a that the maximum stress of drainage asphalt mixtures prepared by rubber powder-modified asphalt at low temperatures shows a decreasing and then an increasing trend as the temperature rises, and the compressive strength is the largest at −15 °C, with an average compressive up to 3.44 kN, the smallest compression strength at −10 °C, and the minimum stress of 2.36 kN. Figure 8b presents the change of the maximum stress with the temperature of the mixture prepared by SBS-modified asphalt, from which it can be seen that the stress reaches the maximum at −10 °C, and the stress is minimum at −5 °C, which indicates that the drainage asphalt mixture prepared by SBS-modified asphalt has a poor cracking resistance at low temperatures, and is susceptible to fracture at temperatures lower than −10 °C, and is also susceptible to fracture at temperatures lower than −10 °C. This is because the inclusion of SBS changed the cooling amplitude of asphalt. Figure 8c shows the maximum stress of drainage asphalt mixtures prepared by graphene/rubber powder compound modified asphalt as a function of temperature, and the average maximum stress is 3.72 kN at −15 °C, and the average minimum stress is 3.22 kN at −10 °C, which improves low-temperature cracking resistance compared to the rubber powder-modified asphalt with no graphene added. The high- and low-temperature cracking resistance performance was improved by 7.50% and 26.71%, respectively.

Meanwhile, through the comparison of Figure 8a,c, it can be observed that the overall stress change trend of drainage asphalt mixtures prepared by rubber powder-modified asphalt with a graphene addition is relatively smooth, and the degree of the undulation of the crack resistance of the mixtures is small, and the low-temperature crack resistance is more excellent.

### 3.4. Low-Temperature Cracking Resistance After Freeze–Thaw Cycling

**Figure 9:**
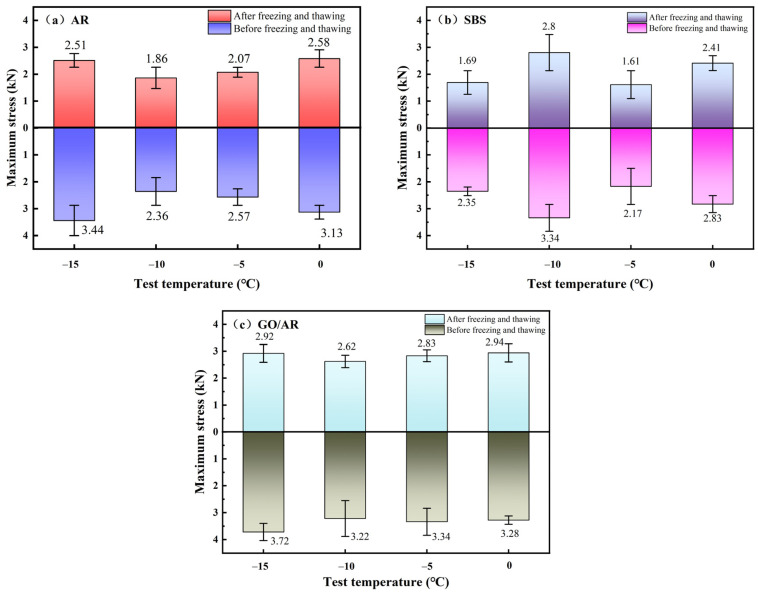
A comparison of the maximum stress before and after freeze–thaw cycles: (**a**) Maximum stress before and after AR freeze-thaw cycle. (**b**) Maximum stress before and after SBS freeze-thaw cycle. (**c**) Maximum stress before and after GO/AR freeze-thaw cycle.

For the actual application of pavement, the low-intensity cracking resistance of graphene/rubber powder compound modified asphalt is tested by the pressure test after freezing and thawing on the standard semicircular specimen, and the experiment results are shown in Figure 9.

The maximum stresses were compared before and after freeze- and thaw-circulation for the standard semicircular test in Figure 9. All data are based on three sets of independent parallel tests (n = 3), and the error bar is clearly marked as the standard deviation. It is evident from Figure 9a–c that the cracking strength of the SCB specimens decreased after the freeze–thaw circulation, and the maximum stresses that they could withstand all decreased compared with those of the SCB specimens that did not undergo the freeze–thaw circulation. For the SCB specimens prepared from the rubber powder-modified asphalt in Figure 9a, the maximum stress decreased by 27.03% at −15 °C and 17.57% at 0 °C, and the overall trend is the same as that of the SCB specimens that have not been subjected to freezing and thawing, while for the SBS-modified asphalt in Figure 9b, the maximum stress decreased by 28.09% at −15, and 14.84% at 0 °C, but at −5 °C, the overall trend shows that the maximum stress decreases first and then increases, contrary to the unfrozen and thawed SCB specimens, indicating that the crack resistance of drainage asphalt mixtures prepared by SBS-modified asphalt is unstable at low temperatures. As can be seen in Figure 9c, graphene/rubber powder compound modified asphalt has a maximum stress decrease of 21.51% at −15 °C compared to that of the frozen and thawed SCB specimen, and a decrease of 10.37% at 0 °C, with a minimum degree of 10.37%, with the smallest degree of 10.37%. Compared with the SCB specimens of the drainage asphalt mixture prepared with rubber powder-modified asphalt, the inclusion of graphene slowed down the maximum stress decrease by 5.52% at −15 °C, and 7.2% at 0 °C, and the degree of slowdown increased with the increase of temperature, and the inclusion of graphene strengthened the low-thermal crack-resistant performance of rubber powder-modified asphalt.

**Figure 10:**
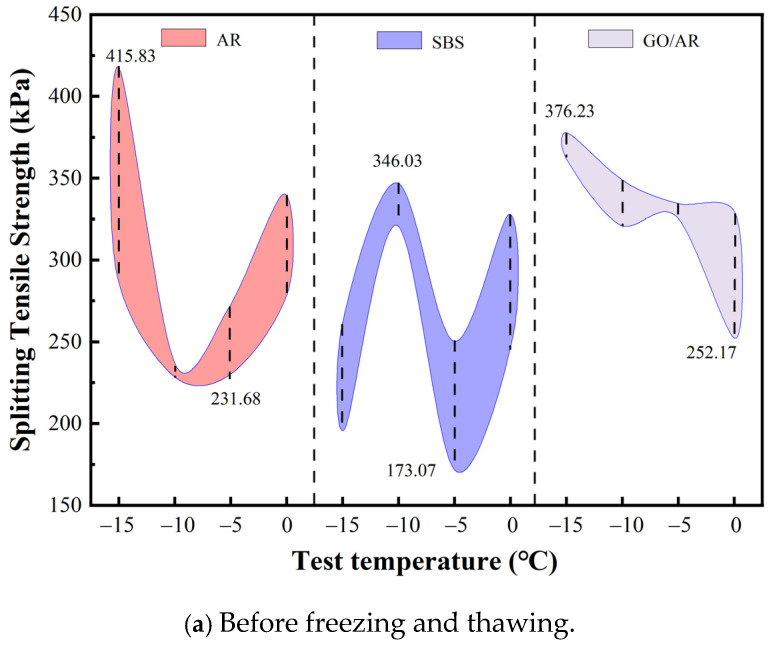

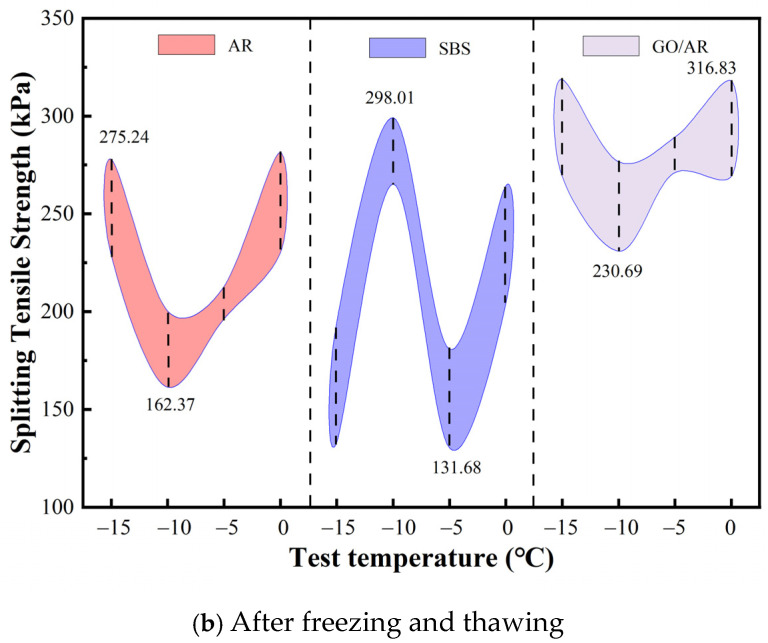
The range of the splitting tensile strengths of semi-circular specimens before and after freeze–thaw cycles.

The splitting tensile strengths of the three modified asphalts at different temperatures are shown in Figure 9a and Figure 9b, respectively. Figure 9a shows the range of splitting tensile strengths before freezing and thawing cycles, and Figure 9b shows the range of splitting tensile strengths after freezing and thawing cycles. As can be seen in Figure 10a, the range of the split tensile strength of drainage asphalt mixtures prepared with rubber powder-modified asphalt is between 415.83 −231.68 kPa at −15 °C to 0 °C, and the range of the split tensile strength of drainage asphalt mixtures prepared with SBS-modified asphalt is between 346.03–173.07 kPa at −15 °C to 0 °C. The graphene/rubber powder compound modified asphalt-prepared drainage asphalt mixture is at −15 °C to 0 °C, with a split tensile strength range between 376.23–252.17 kPa. from Figure 10b, it can be seen that the rubber powder-modified asphalt-prepared drainage asphalt mixture is at −15 °C to 0 °C, with a split tensile strength range between 275.24–162.37 kPa, and the split tensile strength of drainage asphalt mixtures prepared by SBS-modified asphalt ranged from 298.01–131.68 kPa at −15 °C to 0 °C, and the split tensile strength of drainage asphalt mixtures prepared by graphene/rubber powder compound modified asphalt ranged from 316.83–230.69 kPa at −15 °C to 0 °C.

The comparison of split tensile strength ranges in Figure 10a,b shows that, as follows: the split tensile strength of the drainage asphalt mixture prepared by rubber powder-modified asphalt decreased by 209.9 kPa before and after the freeze–thaw cycle, the split tensile strength of mixture prepared by SBS-modified asphalt decreased by 89.41 kPa after the freeze–thaw cycle, and the mixture prepared by graphene/rubber powder compound modified asphalt decreased by 80.88 kPa after the freeze–thaw cycle. Compared with three kinds of modified asphalt, the split tensile strength of the mixture prepared by graphene/rubber powder compound modified asphalt decreased by 80.88 kPa at low temperatures. The splitting tensile strength of the graphene/rubber powder compound modified mixture decreased by 80.88 kPa before and after the freeze–thaw cycle.

## 4. Graphene/Rubber Powder Composite Modified Asphalt Under Microstates

**Figure 11:**
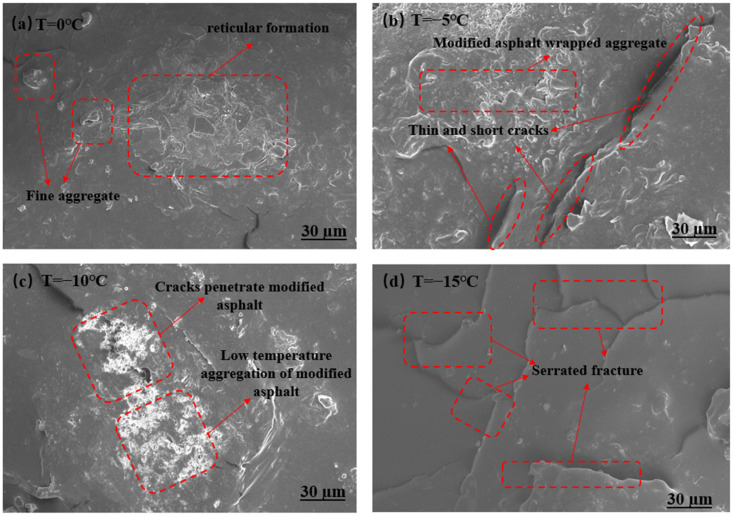
The microscopic morphology of permeable asphalt mixtures at different temperatures: (**a**) Microscopic image at 0 °C. (**b**) Microscopic image at −5 °C. (**c**) Microscopic image at −10 °C. (**d**) Microscopic image at −15 °C.

The dispersion of graphene in rubber powder-modified asphalt affects the low-temperature performance of the modified asphalt in drained asphalt mixtures, and scanning electron microscopy (SEM) was used to observe the dispersion of rubber powder-modified asphalt and graphene/rubber powder-modified asphalt in the microscopic state [42], as shown in Figure 11.

As shown in Figure 11a–d, we can see the microscopic morphology of permeable asphalt mixtures prepared by graphene/rubber powder composite modified asphalt at 0 °C, −5 °C, −10 °C, and −15 °C, respectively. When rubber powder was mixed in asphalt, the high-modulus and high-elasticity properties of rubber powder in asphalt were maintained, and the performance of rubber asphalt in terms of shear deformation resistance and deformation recovery was significantly enhanced compared with that of matrix asphalt. However, the intercalation between rubber powder and asphalt is relatively poor, and the contact surface is relatively small, which indirectly affects the viscoelasticity between rubber powder-modified asphalt and aggregate. The addition of graphene dispersed the aggregation situation of rubber powder, dispersed the irregular net-like aggregation morphology of rubber powder into asphalt, and the rubber particles were reduced, which optimized the intercalation situation between rubber powder and asphalt, increased the connection area between graphene/rubber powder and asphalt, and increased the degree of adhesion between graphene/rubber powder composite modified asphalt and aggregate.

As can be seen from Figure 11a, the graphene flake layer and rubber powder particles are dispersed more uniformly in the asphalt matrix at 0 °C, forming a continuous mesh reinforced structure. The pore distribution is uniform, and the interface between asphalt mortar and aggregate is tightly combined without obvious cracks or defects. At this temperature, the elastic deformation capacity of rubber powder is dominant, which can effectively absorb small stresses, and the high modulus of graphene can limit the local deformation through the role of “bridging” to delay the emergence of cracks. As can be seen from Figure 11b, showing −5 °C localized areas of short cracks, cracks start from the edge of the aggregate or pore space. Graphene flakes appear slightly agglomerated, but still with the rubber powder to form a crosslinked structure, and low-temperature asphalt mortar brittleness increases, the stress is concentrated in the pore or aggregate interface, and the crack deflection effect of graphene and the elastic dissipation of the rubber powder can still partially inhibit the crack expansion. From Figure 11c, it can be seen that the number of cracks under −10 °C increased significantly, and some of the cracks occurred through the graphene/rubber powder composite modified asphalt. Pore edges appear in a “jagged” fracture morphology, the asphalt matrix rigidity is dominant, it has a rubber powder elasticity failure, the graphene enhancement effect is limited by its low-temperature compatibility with the matrix, and the crack extension energy exceeds the composite material load-bearing threshold. From Figure 11d, it can be seen that −15 °C presents a large-scale continuous crack network, graphene lamellae are pulled off or peeled off, and the rubber powder particles are separated from the matrix to form holes. The overall structure is loose, and the permeable pores are connected by the cracks. The composite material undergoes brittle fracture at extremely low temperatures, and the reinforcing effect of graphene and the toughening mechanism of rubber powder fail, and the cracks expand rapidly along the stress concentration area.

The low-temperature cracking resistance of graphene/rubber powder composite asphalt showed significant temperature sensitivity. In the range of 0 °C to −10 °C, the composite material maintains the crack resistance through the synergistic mechanism of “graphene crack arresting + rubber powder energy dissipation”. Below −10 °C, the synergistic mechanism fails, and the crack extension dominates the damage. Below −10 °C is the critical temperature for the crack resistance of the composite material, which is related to the acceleration of the glass transition of the asphalt matrix and the debonding of the modifier/matrix interface. The pores of the permeable asphalt became a source of stress concentration at low temperatures, but the incorporation of the modifier slowed down the rate of crack initiation at the pore edges, indicating that the permeability function and the crack resistance can be partially compatible by microdesign. Enhancing the dispersion of graphene and improving the low-temperature compatibility of rubber powder and asphalt are the keys to further broaden the applicable temperature range of this material.

## 5. Conclusions

In the present paper, the low-intensity cracking resistance of graphene/rubber powder compound modified asphalt at different temperatures was investigated and reached the following conclusions:

(1) Under the condition of −15–0 °C, the magnitude of the fracture energy of drainage asphalt mixtures prepared by graphene/rubber powder compound modified asphalt was proportional to the temperature, and the inclusion of graphene improved the fracture energy by 15.68% of rubber powder-modified asphalt, which significantly slowed down the decrease of the fracture energy and improved low-intensity cracking resistance, as well as reduced the range of the mixture’s decreasing fracture energy with the decrease of the temperature.
(2) When compared to rubber powder-modified asphalt, graphene/rubber powder compound modified asphalt improved the maximum stress by 7.50% and 26.71% at −15 °C and −10 °C, respectively. The introduction of graphene resulted in smoother stress changes in the mix at different temperatures, which improved the low-intensity cracking resistance and showed a superior low-intensity cracking resistance.
(3) The crack strength of SCB specimens generally decreased after a freeze–thaw cycle. Rubber powder-modified asphalt specimens showed a decrease of 27.03% and 17.57% in maximum stress at −15 °C and 0 °C, and the trend was the same as that of the unfrozen and thawed specimens. SBS-modified asphalt specimens showed a decrease of 28.09% in maximum stress at −15 °C and a decrease of 14.84% at 0 °C, but a phenomenon of decreasing and then increasing at −5 °C, which showed the instability in low-temperature cracking resistance. The maximum stress of graphene/rubber powder compound modified asphalt specimens decreased by 21.51% and 10.37% at −15 °C and 0 °C, and the inclusion of graphene significantly slowed down the degree of decrease in the maximum stress compared with that of rubber powder-modified asphalt, and the decrease in split tensile strength was the smallest after the freeze–thaw cycle, and the inclusion of graphene improved the low-intensity cracking resistance at low temperatures.
(4) In the microscopic state, the addition of graphene dispersed the aggregation of rubber powder, dispersed the irregular mesh aggregation morphology of rubber powder more uniformly into the asphalt, enhanced the intercalation of rubber powder and asphalt, and increased the connecting area between graphene/rubber powder and asphalt, and the low-temperature cracking resistance of graphene/rubber powder composite modified asphalt had a significant temperature-dependent property, with a critical temperature point of −10 °C. In the range of 0 °C to −10 °C, the crack resistance is maintained through the synergistic effect of graphene and rubber powder, while when the temperature is lower than −10 °C, the glass transition of the asphalt matrix and interfacial debonding are intensified, leading to the failure of the synergistic mechanism, and the cracks expand rapidly to dominate the damage.

## Figures and Tables

**Table 1:** Main technical indicators of 70 # base asphalt.

Technical Indicators	Test Result	Technical Requirement
Penetration/(100 g, 25 °C, 5 s)/0.1 mm	66.9	60~80
Softening point/°C	48.3	≥46
Ductility/(15 °C, 5 cm/min)/cm	>100	≥15
Flash point	277	≥269

**Table 2:** The main technical indicators of graphene.

Purity/%	Layer Size/μm	Number of Layers	Oxygen Content/%	Sulphur Content/%	Specific Surface Area/m^2^/g
>95	5–50	6–10	0.5	0.5	100–300

**Table 3:** Basic indicators of basalt mineral materials.

Technical Indicators	Test Result	Technical Requirement
Crushing value/%	11.2	≤26
Los Angeles wear value/%	10.7	≤28
Water absorption rate/%	0.4	≤2
SiO_2_ content/%	56.38	/
Needle like particle content/%	7.33	≤15

**Table 4:** Three conventional properties of modified asphalt.

Technical Indicators	AR	SBS	GO/AR
Penetration/(100 g, 25 °C, 5 s)/0.1 mm	81.4	88.3	92.5
Ductility/(15 °C, 5 cm/min)/cm	69.4	72.3	71.4
Softening point/°C	65.3	52.7	68.4
60 °C Dynamic viscosity (kPa·s)	28.6	54.6	32.4
Actual measured average porosity (%)	20.5	21.4	21.7

## Data Availability

All data supporting the results of this study are included in the article.
